# Supplementary material for: Identification of photoperiod-induced specific miRNAs in the adrenal glands of Sunite sheep (Ovis aries)
Source: Front Vet Sci. 2022 Jul 22;9:888207. doi: 10.3389/fvets.2022.888207 (PMC9354845; doi:10.3389/fvets.2022.888207)
Supplement: Supplementary file 1 [file Data_Sheet_1.ZIP › Supplementary file/miRDeep2 file.zip!/miRDeep2 file/Know_Structure/SP42A2/oar-mir-411b.pdf]

miRBase precursor : oar-mir-411b
Total read count : 3007
oar-miR-411b-5p read count : 2955
oar-miR-411b-3p read count : 50
remaining reads : 2

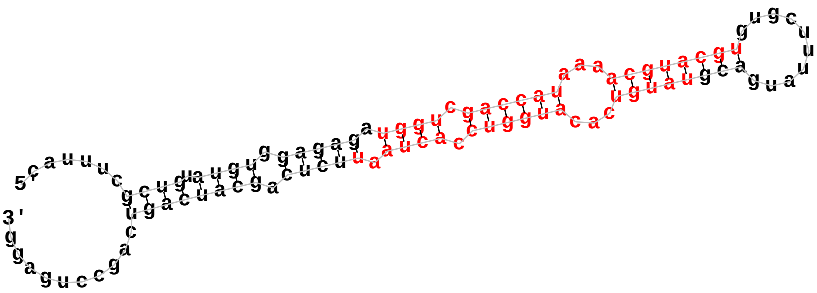

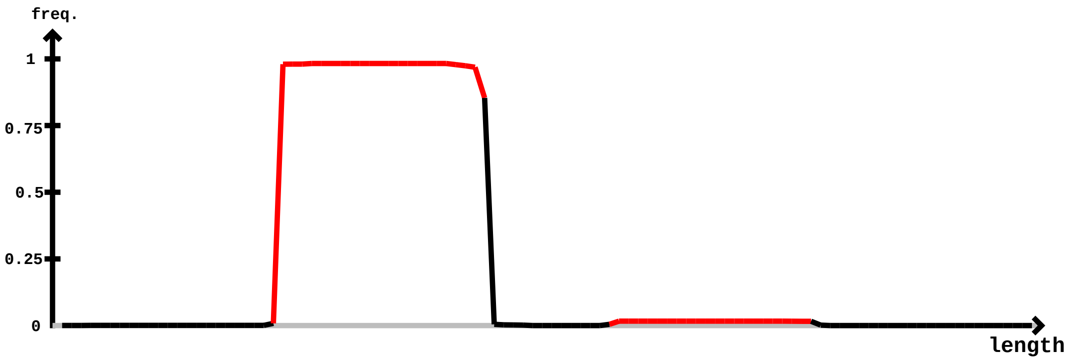

oar-miR-411b-3p

oar-miR-411b-5p

```
5'- cauuucgcuguauguggagagauggucgaccauaaaacguacgugugcuuuaugacguaugucacaugguccacuaauucucagcaucagucagccugagg  -3'    exp
    ......((((.((((.(((((.((((.((((((...((((((((..........))))))))...)))))).)))).))))).))))))))..........   reads   mm   sample
    cauuucgcuguauguggagaga...............................................................................   1       0    seq
    ..uuucgcuguauguggagaga...............................................................................   1       0    seq
    ....................auggucgaccauaaaacguac............................................................   1       0    seq
    ....................auggucgaccauaaaacguacg...........................................................   18      0    seq
    ....................auggucgaccauaaaacguacgu..........................................................   4       0    seq
    .....................uggucgaccauaaaacgu..............................................................   13      0    seq
    .....................uggucgaccauaaaacgua.............................................................   13      0    seq
    .....................uggucgaccauaaaacguac............................................................   15      0    seq
    .....................uggucgaccauaaaacguacg...........................................................   328     0    seq
    .....................uggucgaccauaaaacguacgu..........................................................   2548    0    seq
    .....................uggucgaccauaaaacguacgug.........................................................   6       0    seq
    .....................uggucgaccauaaaacguacgugu........................................................   1       0    seq
    ......................ggucgaccauaaaacguacgu..........................................................   1       0    seq
    ........................ucgaccauaaaacguacgugug.......................................................   2       0    seq
    ........................ucgaccauaaaacguacgugugc......................................................   5       0    seq
    ........................................................guaugucacaugguccacua.........................   1       0    seq
    ........................................................guaugucacaugguccacuaau.......................   12      0    seq
    ........................................................guaugucacaugguccacuaauu......................   1       0    seq
    .........................................................uaugucacaugguccacu..........................   1       0    seq
    .........................................................uaugucacaugguccacuaau.......................   31      0    seq
    .........................................................uaugucacaugguccacuaauu......................   4       0    seq
```
